# High-Precision Skin Disease Diagnosis through Deep Learning on Dermoscopic Images

**DOI:** 10.3390/bioengineering11090867

**Published:** 2024-08-27

**Authors:** Sadia Ghani Malik, Syed Shahryar Jamil, Abdul Aziz, Sana Ullah, Inam Ullah, Mohammed Abohashrh

**Affiliations:** 1 School of Computing, National University of Computer & Emerging Sciences, Karachi 75030, Pakistan; sadiagmalik.m@gmail.com (S.G.M.); abdulaziz@nu.edu.pk (A.A.); 2 College of Computing and Information Sciences, PAF Karachi Institute of Economics and Technology (PAFKIET), Karachi 74600, Pakistan; shahryarcv@gmail.com; 3 Department of Software Engineering, University of Malakand, Malakand 18800, Pakistan; sana.ullahse@uom.edu.pk; 4 Department of Computer Engineering, Gachon University, Seongnam 13120, Republic of Korea; 5 Department of Basic Medical Sciences, College of Applied Medical Sciences, King Khalid University, Abha 61421, Saudi Arabia; mabuhashra@kku.edu.sa

**Keywords:** skin disease, deep learning, convolutional neural network (CNN), support vector machine (SVM), random forest (RF), machine learning

## Abstract

Dermatological conditions are primarily prevalent in humans and are primarily caused by environmental and climatic fluctuations, as well as various other reasons. Timely identification is the most effective remedy to avert minor ailments from escalating into severe conditions. Diagnosing skin illnesses is consistently challenging for health practitioners. Presently, they rely on conventional methods, such as examining the condition of the skin. State-of-the-art technologies can enhance the accuracy of skin disease diagnosis by utilizing data-driven approaches. This paper presents a Computer Assisted Diagnosis (CAD) framework that has been developed to detect skin illnesses at an early stage. We suggest a computationally efficient and lightweight deep learning model that utilizes a CNN architecture. We then do thorough experiments to compare the performance of shallow and deep learning models. The CNN model under consideration consists of seven convolutional layers and has obtained an accuracy of 87.64% when applied to three distinct disease categories. The studies were conducted using the International Skin Imaging Collaboration (ISIC) dataset, which exclusively consists of dermoscopic images. This study enhances the field of skin disease diagnostics by utilizing state-of-the-art technology, attaining exceptional levels of accuracy, and striving for efficiency improvements. The unique features and future considerations of this technology create opportunities for additional advancements in the automated diagnosis of skin diseases and tailored treatment.

## 1. Introduction

The human skin, being the largest organ in the body, is highly vulnerable to direct and indirect exposure to environmental substances. The human skin serves as a barrier that prevents the infiltration of germs. The skin exhibits varying reactions to these substances, contingent upon individual tolerance and thresholds. The human skin offers substantial defense against both transdermal water loss and external factors. Additionally, it functions as an effective regulator of body temperature in both cold and hot climates. The incidence of skin cancer has exhibited a significant upward trend in recent decades. It is more prevalent among those of Caucasian descent, affecting approximately 2.6% (or 1 in 38) of this population, compared to approximately 0.1% (or 1 in 1000) among individuals of brown or black ethnicity [1]. Based on previous cases, it has been noted that skin cancer (melanoma) ranks as the 13th most prevalent cancer in males and the 15th most prevalent cancer in females [2,3,4].

Skin illnesses can be caused by poor food, environmental changes, weak immune systems, UV radiation exposure, and other factors [1,2]. Some skin disorders develop severely and lead to skin cancer because people are unconcerned or never see a dermatologist. Some non-cancerous disorders can become malignant. Being of an older age and family history also matter [1]. Early diagnosis may treat sickness and prevent irreversible decline. Skin cancer, acne, psoriasis, nail infections, eczema, and other skin problems are common. Dermatoscopy, which acts like a microscope and identifies a different pattern on the skin’s surface, can diagnose diseases like scabies, eczema, fungal infections, melanoma, hair loss, nail folds, and more. Undetectable lesions’ color, texture, boundaries, and size can be measured by dermoscopy’s high-resolution photographs [5]. Doctors may biopsy skin lesions to remove their tiny surfaces. This manual technique is painful and time-consuming.

AI and machine learning have helped accommodate huge amounts of data on the internet, which is now public [6]. Many datasets have been generated in recent decades, but few methods have been developed to use them. Now that algorithms are mature enough to use big data, their applications are expanding. AI and machine learning are used in automatic car driving, robotic surgery arms, predictions, language processing, medical image analysis, X-ray analysis, and more. Machine learning and deep learning can simplify skin disease detection [5,6,7,8]. A benchmarked image-based skin disease classification model should be proposed. Thus, this study would help medical firms manufacture automated skin detection devices for customers and provide health practitioners with a technology that can accurately categorize diseases.

Total body skin scanning systems are increasingly gaining attention to screen skin cancer by analyzing the entire body’s skin surface using chunks of photos by using a digital device that rotates around the whole body and has high-resolution cameras attached to capture images. This helps in the early diagnosis of skin diseases, and the chances of curability increase [9,10,11,12,13].

The remaining article is organized as follows: In Section 2, the related contributions are described briefly with our approach of picking relevant papers. In Section 3, details of datasets are elaborated on in terms of data preparation, and different methods are proposed. Section 4 defines our research experiments and results with a precise discussion. Section 5 is used to conclude the study and share some of the future considerations that can be applied to extend this research.

The current research on skin illness has identified several gaps in knowledge. Previous studies have mostly focused on addressing the binary issue of whether a skin condition is cancerous or not, leaving other aspects of skin disease relatively unexplored. However, they have also neglected to consider numerous other disorders that could potentially contribute to the development of cancer. The primary challenge in this domain is the resemblance between different classes, which might potentially be addressed by implementing ABCDE rules. Nevertheless, the primary emphasis of the present investigation is on the classification of several classes, specifically malignant, benign, and basal cell carcinoma. 

This study’s contributions are as follows:

- A Computer Assisted Diagnosis (CAD) framework has been developed to detect skin illnesses at an early stage.
- We suggest a computationally efficient and lightweight deep learning model that utilizes a CNN architecture.
- The study has contributed by providing brief details on related research on the existing problem.
- The study has contributed by comparing shallow learning and deep learning models.
- Data augmentation is the most important measure in the classification of skin diseases, as this knowledge has been generated by performing different experiments.

## 2. Literature Survey

### 2.1. Methodology

The literature review process has been inspired by the PRISMA technique, which involves the inclusion and exclusion of some research papers.

Initially, I searched the Google Scholar site using the keywords “skin disease, machine learning, deep learning, skin lesion detection, convolutional neural network, SVM, random forest, shallow learning, hybrid, ensemble”. After the initial search result, I scoped multiple papers by filtering them to within the last five years. 

After a quick review of the abstract and conclusions, papers related to skin disease were excluded; moreover, papers published on unknown sites were also excluded. Furthermore, searching was performed by obtaining keywords from specific papers selected initially; however, after this collection, additional papers were excluded based on irrelevant methodologies. The papers that were picked for our research have been included based on the criteria of having a relevant abstract, conclusions, and methodology.

### 2.2. Related Work

In other research, a system was designed to detect two types of skin disease, malignant and benign, by using ANN and SVM. These two machine-learning models took PH2 dataset images as an input after applying pre-processing steps of segmentation and feature extraction/selection. The study featured an accuracy of 94%, a precision of 96%, and a recall of 92%. The authors also utilized the adaptive snake (AS) algorithm and region growing (RG) algorithms for automated segmentation, in which AS was more accurate compared to RG in terms of accuracy. They obtained values of 96% and 90%, respectively [12,13].The study presented in one paper focuses on an adaptive federated approach by implementing a cloud server for connecting a global and local model. Each local model has a learning classifier, and new data are given for training. After training, all local models update their weights and transfer them to the global model, which acts as a primary node to update its weight. A continuous update process runs during image analysis at the end of doctors’ reviews. This has been implemented using the ensemble technique, and, by using this approach, a constant improvement is observed. The model performs better and better when new data are chipped in. The clustering-based mechanism is implemented for segregating different classes, which are like features of skin disease [14,15,16].

The literature also has a case where skin lesion artifacts are considered noise in images, which must be removed before training in the pre-processing stage. The author proposed K-means clustering for the segmentation of images before training in the Mask R-CNN model. This model was selected because of its super-pixel selection feature. The public dataset of the ISIC was used for the experiment, which has unbalanced images [17].

In another study, the CNN model was implemented for multi-class classification by two types of approaches. The first model, which was only a single CNN model trained on the HAM10000 dataset, resulted in an accuracy of 77%. The second model, a combination of multiple CNN models intersecting in a pairwise manner with C1 in an ensemble fashion, resulted in an accuracy of >90%. As a pre-processing step, only standardization was carried out on ranges of [0–1] before training, whereas data expansion was carried out by duplicating by rotating, scaling, translating, and adding noise [18].

Separate research features a proposed approach based on a convolution neural network (AlexNet), where the DermNet database is used for input images. The paper mainly focuses on limited skin diseases, i.e., acne, keratosis, and eczema herpeticum, and each class contains 30 to 60 samples. The learning model has been used with an activation layer, max pooling layer, fully connected layer, and softmax activators, resulting in accuracies of 85.7%, 92.3%, 93.3%, and 92.8% for acne, keratosis, eczema herpeticum, and urticaria, respectively [19,20].

In one particular study, CAD systems were scaled by training four deep learning networks and achieving the best accuracy levels on the DermNet dataset by using disease taxonomy and the ISIC-2018 dataset with seven classes. A model was trained on a pre-trained ImageNet database and a fine-tuned model for dermatological images on ResNet-152, NASNet, DenseNet-161, SE-ResNetXt-101, and, lastly, four models were combined into an ensemble to produce the result with the best accuracy. Training and testing data were divided by using the K-Fold technique as a pre-processing step. Images were cropped and flipped randomly. The class imbalance issue was catered to using the weighted loss approach [21].

Another paper shows an experiment with a deep CNN using three datasets for training a lightweight model with a less computational cost for classifying melanoma disease. Traditional pre-processing steps were followed by normalizing data by converting images into grayscale, making them lower resolution, and cropping images. As public data are imbalanced in terms of different classes, some data augmentation was required; hence, the author implemented this by rotating, scaling in XY directions, and translating by −5 and +5. The augmented data were supplied only for training. In contrast, the validation and test sets were the same as those in public datasets, which are the ISIC 2016, 2017, 2020, and PH2, used only for two classes, melanoma and benign. The proposed model (LCNet) contains 31 convolutional layers [22,23].

In a different study, the authors experimented with different non-linear activation functions for the CNN model for its hidden layers, such as ReLU, Elu, Clipped ReLU, LeakyReLU, PReLU, and Tanh. The proposed model of a CNN was trained with six different activation functions. Specifically, a small dataset of PH2 and 300 images from the ISIC database were used for training a model for only three classes: melanoma, benign, and nevus. To handle the issue of class imbalance, some data augmentation techniques were implemented by rotating images 180° The LeakyReLU outperformed the remaining functions for melanoma and Clipped ReLU did for the remaining two diseases [24].

In a separate paper, a metric-specified loss function was proposed, inspired by the kappa index, and a comparison with the Dice index was made with the introduced loss function known as the kappa loss. It took all the pixels from the image, including true negatives. The experiments were carried out on six public datasets of melanoma and non-melanoma cases for making a two-class classification on a U-Net state-of-the-art model, with a total of 31,031,685 parameters. The model was evaluated with two different loss functions: Dice and kappa. The Hausdorff distances were calculated for these similar loss functions, and the experiment shows that kappa converges faster than Dice. Hence, this study offered a new means of skin segmentation for the better training of models in cases of removing skin artifacts [25].

In a further study, they used clinical images of pigmented skin lesions taken from digital cameras and specifically worked on malignant and benign diseases. The training and testing data were split as 70% and 30% of the given data, respectively. The proposed model, Faster R-CNN, was compared with ten BCDs and ten dermatological trainees (TRNs) on six-class and two-class classifications in which FR-CNN was proved to result in the best accuracy, an accuracy greater than 85%. The parameter settings featured VGG-16 as the backbone and SGD for the optimizer and performed 100 epochs. Some data augmentation techniques were also implemented, such as flipping the image, cropping, zooming, and rotating [26].

A separate paper proposed a new classification process using pre-trained models such as VGG16, Inception, Xception, MobileNet, ResNet50, and DenseNet161. They selected the top four models for targeted ensemble classification. All these models were tested on the HAM10000 dataset for all seven skin conditions. The experiment was performed with a ratio of 6:3:1 of the data available in the selected dataset. The pre-processing step was performed before getting into the core of the proposed model, which included color featuring, model transfer, and data balancing. Moreover, they undertook a cross-validation analysis. They used a classic voting system for four models, which took as its input an image and resulted in the predicted class, and initial voting was given to all model results with equal weights, and a pre-trained CNN model was be used for the investigation of the correctness of the result. If the model was nominated before pruning, then it would be subjected to a thorough evaluation. Otherwise, a new image was sent into the ensemble, and the classification process was started from the initial stage.

Furthermore, an IoT mobile device was used to generate data and send it to Fog Layer, which is responsible for transferring image data to an AI-based classification approach. The result was then transferred to a specialist’s machine, and feedback was taken in the meantime to better the accuracy of training [27].

In another study, the main objective of this research was to present a hybrid approach to classify skin lesions in binary classification patterns; for example, melanoma vs. all and seborrheic keratosis vs. all. The authors used three pre-trained state-of-the-art models for extracting features: AlexNet, VGG-16, and ResNet-18. In the second stage, the SVM classifier was used for each state-of-the-art model to train on extracted features in the previous stage, and for the final step, the output was fused for classification. The AUC obtained by the experiment was 83.83% and 97.55% for melanoma and seborrheic keratosis, respectively [28].

Other research mainly focused on classifying a subtype of melanoma cancer, acral melanoma, by using a deep learning technique using a seven-layered deep CNN model. The dataset used for training the model was obtained from Yonsei University Health System. The best accuracy which was observed was more than 90%. Moreover, the proposed model was compared with pre-trained models AlexNet and ResNet-18. The whole training process was completed in three stages: stage 1—preprocessing, which included resizing images and removing artifacts using cropping and image processing approaches. Data augmentation was applied to increase data size from 724 to 4344 by flipping and rotating. The average accuracy obtained by the proposed model was 91.03% [29].

A further study took advantage of pre-trained CNN models ResNet50 and VGG16. It combined them to achieve the objective of correctly classifying types of skin diseases into nine categories, which were pigmented keratosis, melnoma, vascular lesion, actinic keratosis, squamous cell carcinoma, basal cell carcinoma, seborrheic keratosis, dermatofibroma, and nevus. The authors designed their methodology in three stages. The first stage was data preparation, which involved data normalization, resizing, color normalization, segmentation, and data augmentation for resolving class imbalance problems. Secondly, the model preparation included the combination of two pre-existing networks in which an image was treated as a raw input. The final stage was to analyze the model by different metrics such as accuracy, precision, recall, and F1-score, which showed that that the hybrid model achieved a precision score of 97.60%, a recall score of 97.55%, and an F1 core of 97.58% [30,31,32].

## 3. Materials and Methods

### 3.1. Datasets and Splitting

The dataset recommended for this framework was one of dermoscopic skin lesion images; these are images taken by a device, like a microscope, to diagnose skin disease type. Dermoscopic images have been used from a challenging dataset from the International Skin Imaging Collaboration (ISIC), which maintains data on susceptible skin lesions. 

The data have been extracted from Kaggle, a collection of ISIC datasets for melanoma and benign lesions. The study is focused on multiclass classification instead of binary class classification; hence, basal cell skin lesion images were collected from the ISIC archive. 

The dataset contained 1197 malignant (Mal) and 1440 benign (Ben) images of a 244 × 244 resolution, whereas the number of basal cell carcinoma (bcc) images was 1125. The imbalanced training set consisted of a total of 3762 images, whereas the test set contained 1060 images, as briefly described in Table 1. The data were highly imbalanced due to the down-sampling performed in Mal and Ben’s class. Hence, each class contained 1125 dermoscopic images. The total balanced training set consisted of 3375 images. Of the training samples, 80% were used for model training, whereas 20% of samples were used for validation for the tuning model, as briefly described in Table 2. However, the test set has been used to measure the model’s performance.

For our experiments, the classes were labeled with 0, 1, and 2 for benign, malignant, and basal cell carcinoma, respectively.

### 3.2. Data Preprocessing

**Figure 1:**
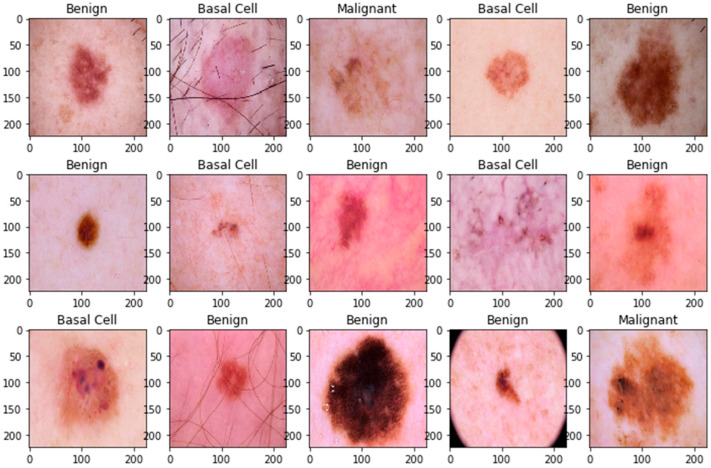
Sample skin lesion images before rescaling operation.

**Figure 2:**
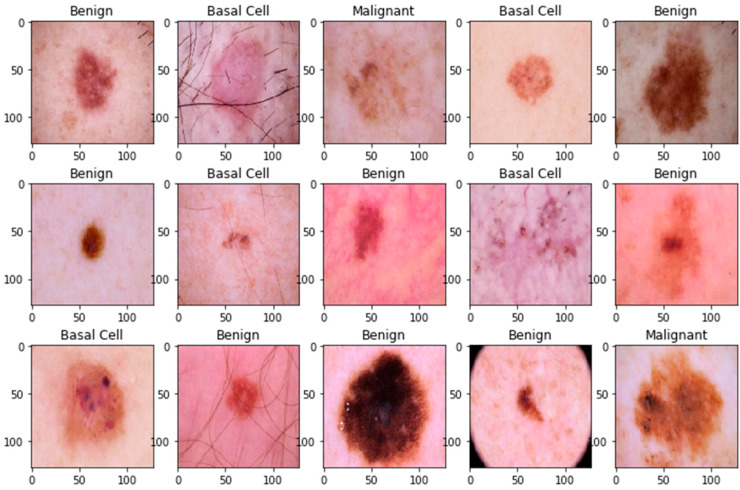
Sample skin lesion images after the rescaling operation.

The pre-processing step was applied to the whole dataset, where a standard resizing of images took place. The images with dimensions of 244 × 244 were reduced to a size of 128 × 128 to lighten the model’s processing, as shown in Figure 1 and Figure 2. There was no need to remove artifacts from original dermoscopic images, such as gel bubbles, ruler marks, hair, etc., as this is not a concern in the current study. The proposed model opted to work with raw images without the elimination of artifacts. Figure 1 illustrates the sample skin lesion images before the rescaling operation.

### 3.3. Data Normalization

The images given to the model for training were normalized by unifying the pixels and dividing their values by 255. Hence, each pixel value was represented on a scale of 0 to 1.

### 3.4. Data Augmentation

The initial dataset was imbalanced due to how the model can be biased to any one disease. Hence, data augmentation has been carried out to increase the model’s performance. Data augmentation has been performed only on training sets locally by using the Keras library, which included flipping an original image left to right, right to left, and rotating 180 degrees. In contrast, no augmentation is performed on test sets. Altogether, this has created a total training set of 13,500 images. However, all experiments were carried out with different cases of imbalanced data with/without augmentation and balanced data with/without augmentation.

### 3.5. Proposed Framework

#### 3.5.1. Basic CNN Model

**Figure 3:**
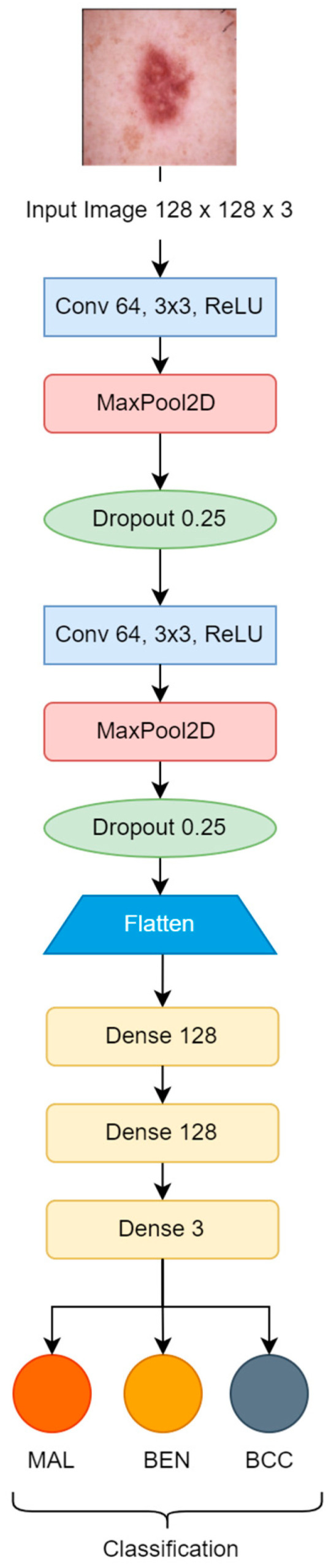
Basic CNN model.

Initially, the convolution neural network model, which is used for training, was constructed from scratch and contains two convolutional blocks with a max pooling layer (2 × 2), and a drop-out factor of 0.25 used in each of them for reducing overfitting. Lastly, three dense layers with 128 units on top of them were activated by the ReLU activation function. The filter size 3 × 3 was used with the same padding in each convolutional layer [25,26]. The Softmax function classified the resulting values as specified labels {MAL, BEN, BCC}, as shown in Figure 3. The Adam optimizer was used for the model’s convergence with a learning rate of “1 × 10^−3^”. Loss was monitored by categorical cross-entropy. The number of total learnable parameters was more than 8 million.

#### 3.5.2. Proposed CNN Model

**Figure 4:**
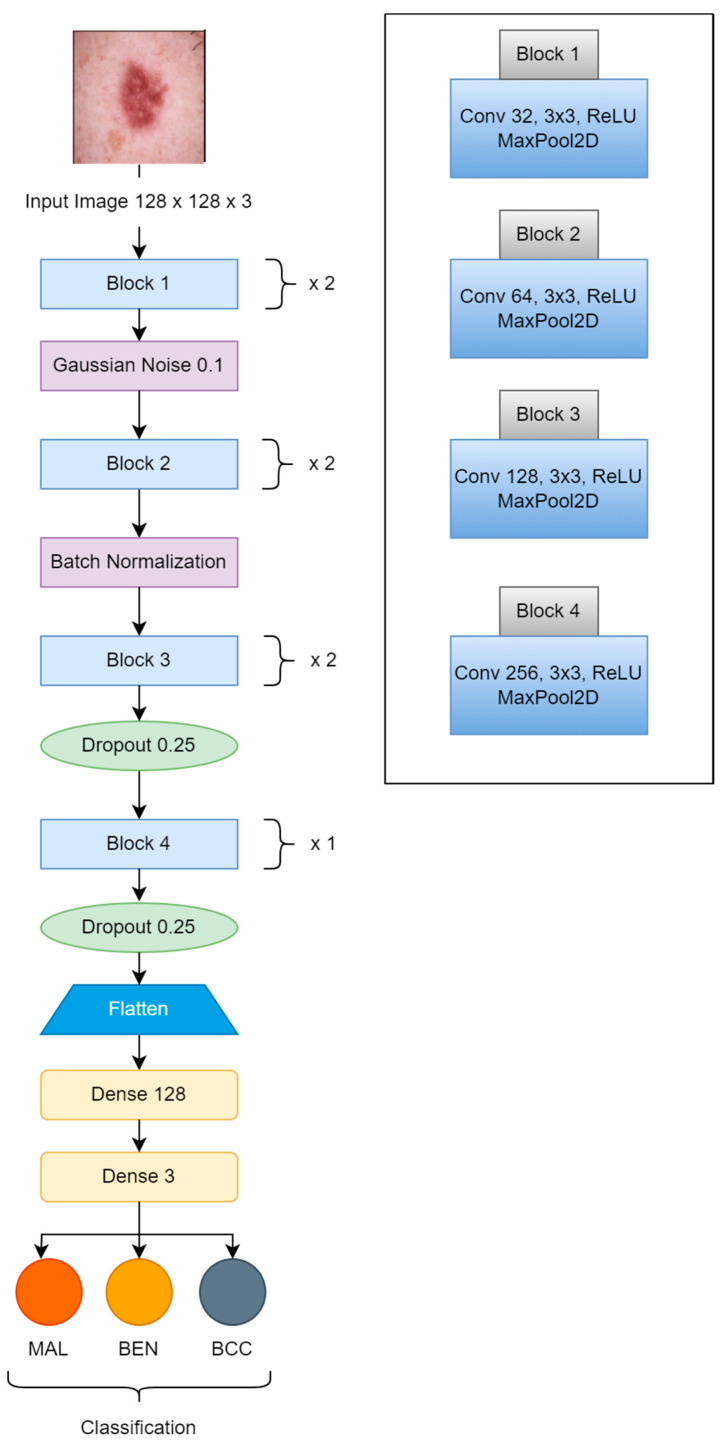
Proposed CNN Model.

The proposed CNN model was implemented with seven convolutional units, using a 3 × 3 kernel size, along with max pooling blocks (2 × 2); gaussian noise was used with a factor of 0.1, and batch normalization and dropout with a factor of 0.25 were also applied to reduce the rate of overfitting. After flattening the outputs, the dense layer with 128 units was activated by the ReLU function, which was transferred to the final dense layer, which was activated by three neurons used for classification by the Softmax activation function as shown in Figure 4. The Adam optimizer was used for better learning, with a rate of “1 × 10^−3^”. Categorical cross-entropy was used for loss monitoring. The learnable parameters number approximately 0.6 million. The hyperparameters were selected after multiple attempts of experiments. Figure 4 is the block diagram of our proposed CNN model.

## 4. Experiments and Results

The first step is image preprocessing. After this step, input images are given to the model in the shape of 128 × 128 × 3, where 3 is the number of channels (R, G, B). The hyperparameters are selected manually for CNN, which include the number of epochs, batch size, learning rate, and regularization threshold.

A three-class (Malignant, Benign, Basal Cell) classification is carried out with data augmentation and without data augmentation. Moreover, some shallow learning algorithms (SVM and Random Forest) have also been tested to compare the accuracies of the CNN model. While training, the SVM grid search technique is used for hyperparameter selection, as described in Table 3. The whole implementation is conducted on Jupyter Notebook on a local machine, and some of the experiments are carried out on Google Colab Pro.

A basic CNN model with two convolutional blocks was tested for 50 epochs. A batch size of 32 and a learning rate of 1 × 10^−3^ is used during the model training phase. In contrast, the proposed CNN model with seven convolutional units is implemented using the same parameter settings as the previous model, as described in Table 4.

The accuracy of the basic CNN model without data augmentation is 55.94%, and the accuracy of the basic CNN with data augmentation is 84.71%. The accuracy of the proposed CNN model without data augmentation is 74.90%, the accuracy of the proposed CNN with data augmentation is 86.69%, the accuracy of the SVM without data augmentation is 74.90%, the accuracy of SVM with data augmentation is 84.24%, the accuracy of Random Forest without augmentation is 81.13%, and the accuracy of Random Forest with augmentation is 84.62%. A hyperparameter for Random Forest is “n_estimators = 100”. The best accuracy after 50 epochs was observed from the proposed CNN model, and is 86.88%.

The proposed model has been tested on 100 epochs; hence, the resulting accuracy of each case is 0.7490, 0.8698, 0.8339, and 0.8764 for the following cases: class imbalance without augmentation, class imbalance with augmentation, class balance without augmentation, and balanced class with augmentation, respectively, as described in Table 5.

### 4.1. Hybrid Model

The hybrid model has been implemented to expand our study to present the most accurate model. Initially, feature extraction was conducted via the proposed CNN model by employing balanced data with augmentation; this approach was selected because it had the highest test accuracy compared to other methods; refer to Table 6. Furthermore, classification is performed by Random Forest with different estimators. The reason for selecting Random Forest with the CNN model is that the test accuracies are higher in all approaches than the SVM, as shown in Table 6.

Table 7 describes the accuracies at different stages of estimators that were manually selected while experimenting.

### 4.2. Performance Metrics

Precision denotes the percentage of correct positive predictions, whereas Recall represents the percentage of correct predictions within whole datasets or in every case, which is also known as sensitivity. The F1-score is just a harmonic mean of Precision and Recall. Recall is more critical in medical domains than precision. Below are the tables that describe the model’s evaluation metrics.

The calculated performance metrics for our study are described in Table 8, Table 9, Table 10 and Table 11 for basic CNN model, proposed CNN Model, SVM and Random Forest respectively.

Analyzing the performance metrics mentioned in Table 8, Table 9, Table 10 and Table 11 shows that our proposed model surpassed the other approaches used to classify skin lesion images.

### 4.3. Accuracy and Loss

Accuracy and loss are the most important evaluation metrics for measuring the model’s performance on specific datasets. The higher the accuracy and lower the loss, the better the model performance is. Below are the graphs of model accuracy and loss for the basic CNN model and the proposed CNN model with different approaches.

**Figure 5:**
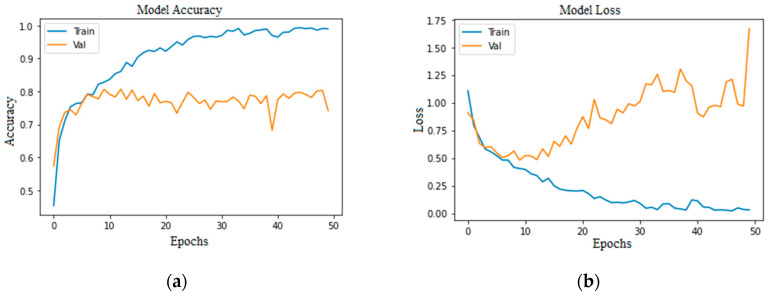

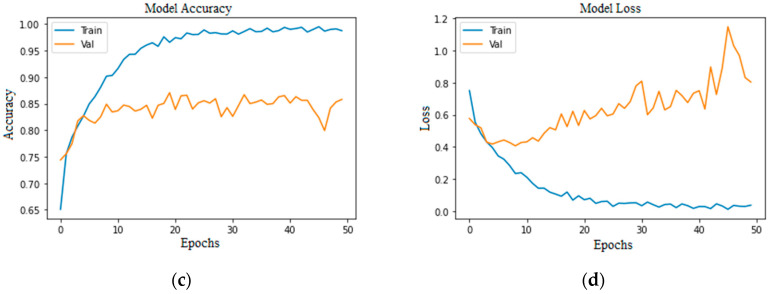
Basic CNN model imbalanced class: (**a**) without augmentation model accuracy; (**b**) without augmentation model loss; (**c**) with augmentation model accuracy; (**d**) with augmentation model loss.

**Figure 6:**
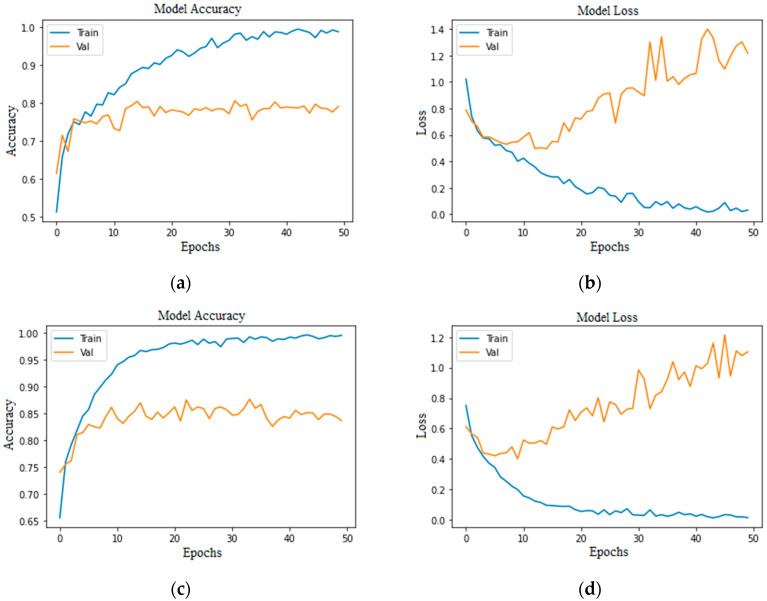
Basic CNN model balanced class: (**a**) without augmentation model accuracy; (**b**) without augmentation model loss; (**c**) with augmentation model accuracy; (**d**) with augmentation model loss.

Figure 5 and Figure 6 show that both training and validation accuracy is increasing, but the loss of training is falling. In contrast, the validation loss started increasing after 15 epochs, which shows that the model has many errors. 

**Figure 7:**
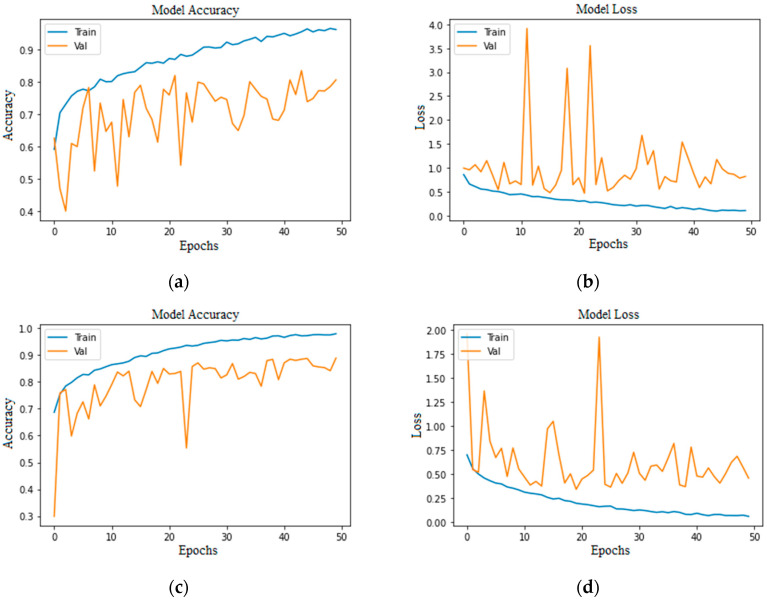
Proposed CNN model imbalanced class (50 epochs): (**a**) without augmentation model accuracy; (**b**) without augmentation model loss; (**c**) with augmentation model accuracy; (**d**) with augmentation model loss.

**Figure 8:**
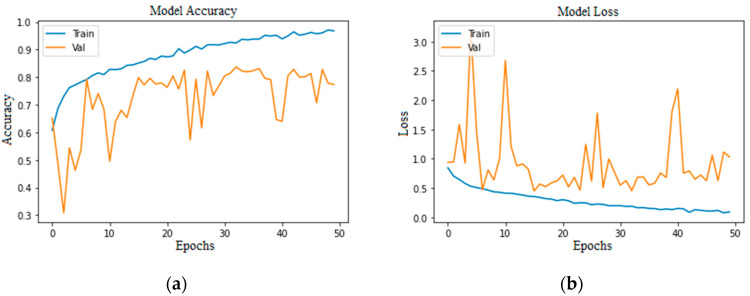

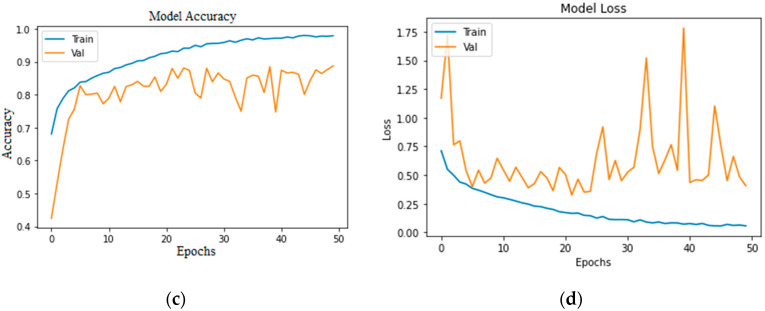
Proposed CNN model balanced class (50 epochs): (**a**) without augmentation model accuracy; (**b**) without augmentation model loss; (**c**) with augmentation model accuracy; (**d**) with augmentation model loss.

As we can see in Figure 7 and Figure 8, the accuracies of training and validation are gradually increasing, and loss is decreasing. Moreover, the validation loss has small gaps with training loss, which indicates a model’s positive learning. However, there are some spikes during the model’s learning process. 

**Figure 9:**
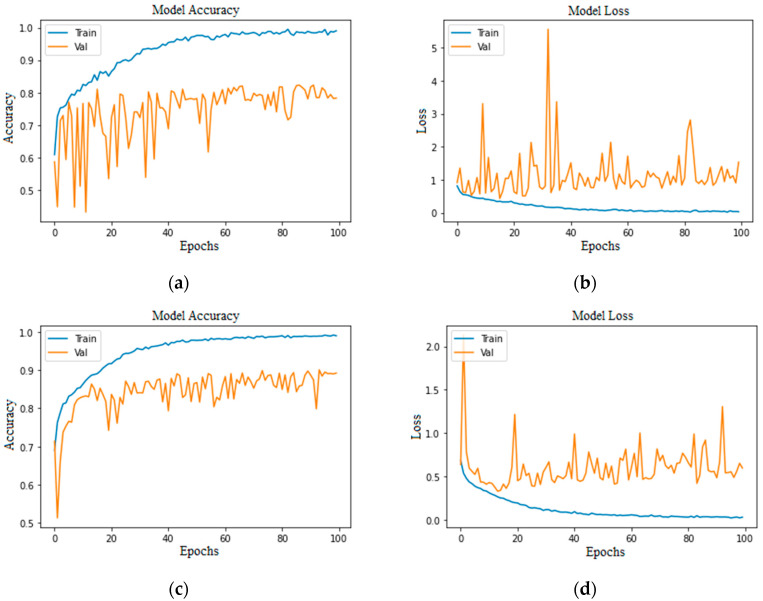
Proposed CNN model imbalanced class (100 epochs): (**a**) without augmentation model accuracy; (**b**) without augmentation model loss; (**c**) with augmentation model accuracy; (**d**) with augmentation model loss.

In Figure 9, the highest training accuracy is 0.9904, whereas the best validation accuracy is 0.80 in Figure 9a and 0.85 in Figure 9c. However, the graphs show a continuous fall in training loss and lots of fluctuations in validation loss, with small gaps in training and validation loss in Figure 9b and a large gap in Figure 9d, which may indicate that a model’s loss started increasing at some point. 

**Figure 10:**
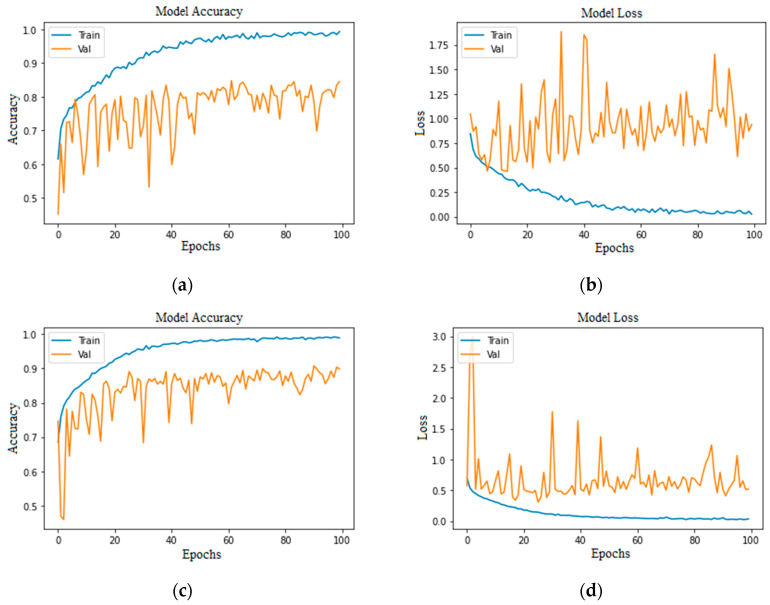
Proposed CNN model balanced class (100 epochs): (**a**) without augmentation model accuracy; (**b**) without augmentation model loss; (**c**) with augmentation model accuracy; (**d**) with augmentation model loss.

In Figure 10a,c, the training accuracy reached its peak with steady increments; on the other hand, loss decreased steadily. In Figure 10b, the training loss is falling, whereas the validation loss started rising after the 20th epoch, which could be a negative in terms of the model’s performance. In Figure 10d the validation loss and training loss are both falling with minimal gaps in between, although there are some peaks in validation loss, but this is understandable while the model is being trained. 

### 4.4. Confusion Matrix

A confusion matrix is another useful metric for evaluating the model’s performance. It specifies the number of true positives, true negatives, false positives, and false negatives.

Confusion matrices define the count of actual vs. predicted in a well-structured format. Actual labels are pre-defined for each sample, and predicted labels are those that a model assigns to each sample as an output, as shown in Table 12. 

Below are figures of our experiment results that visualize skin diseases that are predicted correctly or falsely.

**Figure 11:**
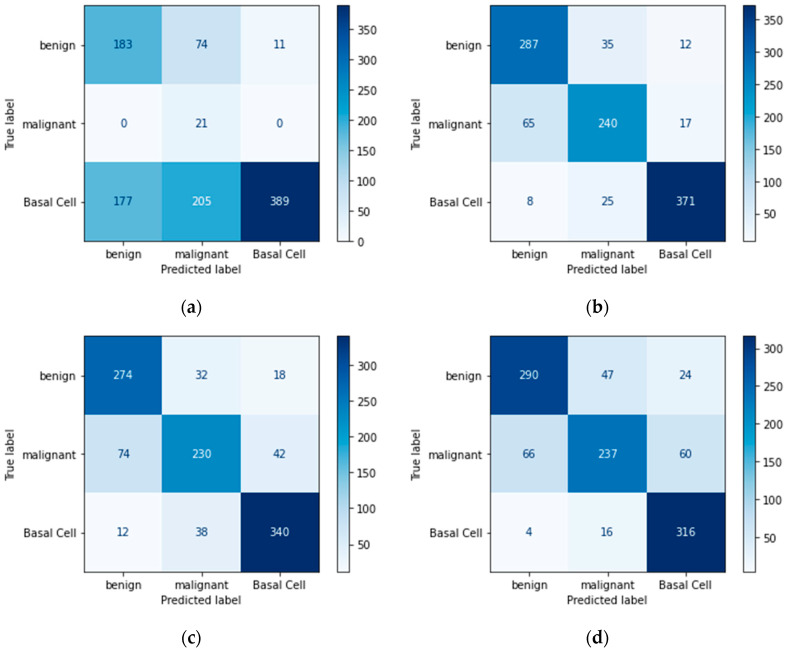
Basic CNN model: (**a**) imbalanced class without augmentation; (**b**) imbalanced class with augmentation; (**c**) balanced class without augmentation; (**d**) balanced class with augmentation.

In Figure 11a, we can see that the prediction of diseases on the test set is not considerable because the model’s prediction has many errors, whereas in Figure 11b–d, the model has predicted more accurate labels for unseen data.

**Figure 12:**
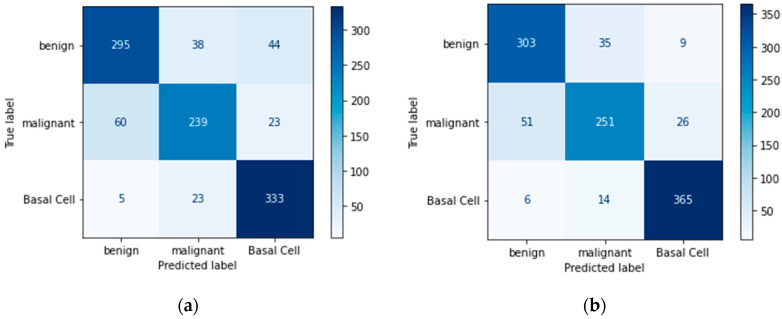

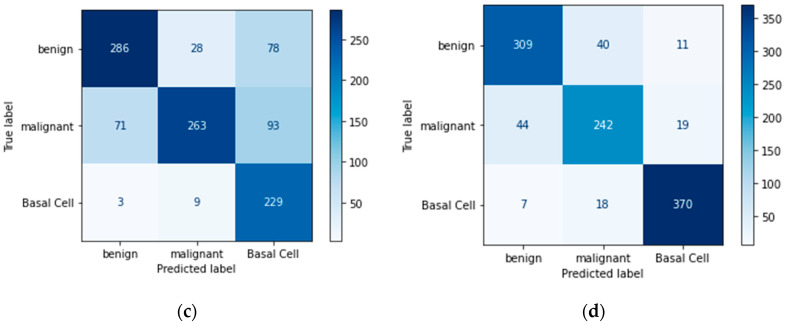
Proposed CNN model confusion matrix (50 epochs): (**a**) imbalanced class without augmentation; (**b**) imbalanced class with augmentation; (**c**) balanced class without augmentation; (**d**) balanced class with augmentation.

In Figure 12a,c, we can see that the experiment’s approach without data augmentation results in poor predictions of diseases on the test set. In contrast, the experiment’s approach with data augmentation creates more accurate predictions, as we can see a proper diagonal in Figure 12b,d.

**Figure 13:**
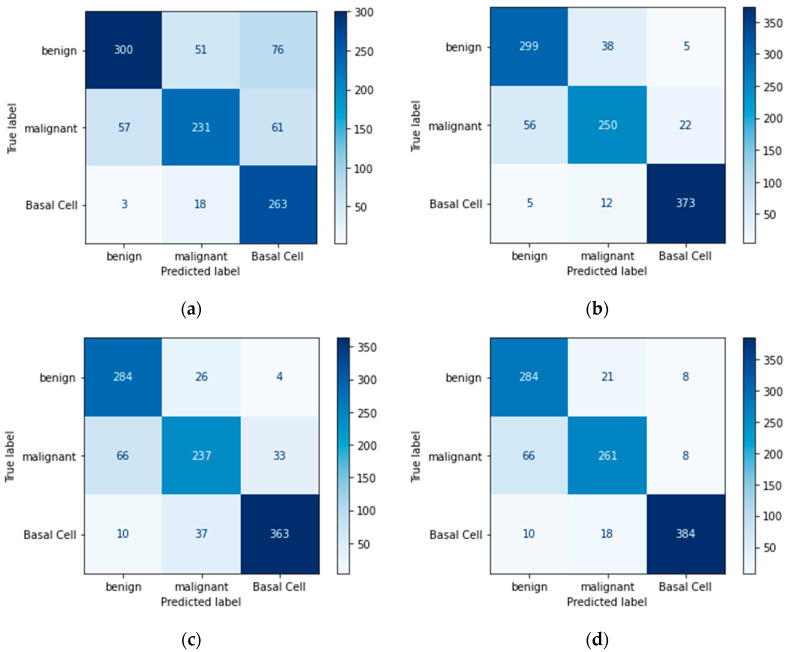
Proposed CNN model confusion matrix (100 epochs): (**a**) imbalanced class without augmentation; (**b**) imbalanced class with augmentation; (**c**) balanced class without augmentation; (**d**) balanced class with augmentation.

Similarly, Figure 13d makes better predictions than the previous experiments. The proposed model has performed up to the mark on the given dataset and can be considered the best model among all comparisons.

As the study continues to compare some shallow learning models for disease classification problems, we can say that the CNN model is more accurate than shallow learning models (SVM and Random Forest). 

**Figure 14:**
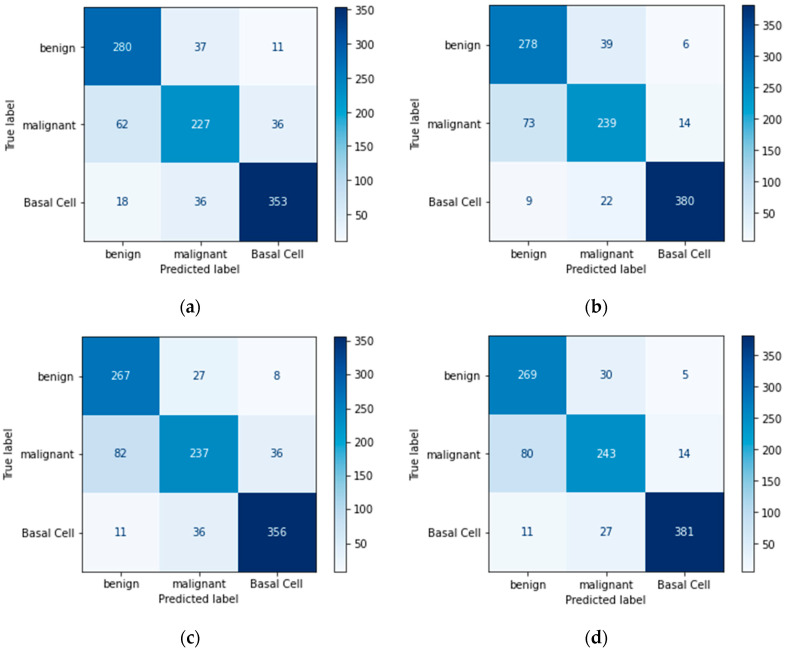
Random Forest confusion matrix: (**a**) imbalanced class without augmentation; (**b**) imbalanced class with augmentation; (**c**) balanced class without augmentation; (**d**) balanced class with augmentation.

In Figure 14a,c, the result of Random Forest without data augmentation has made poor predictions compared to the data augmented approach, as shown in Figure 14b,d.

**Figure 15:**
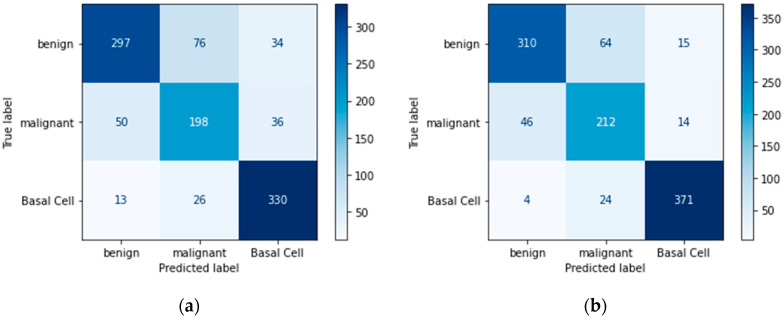

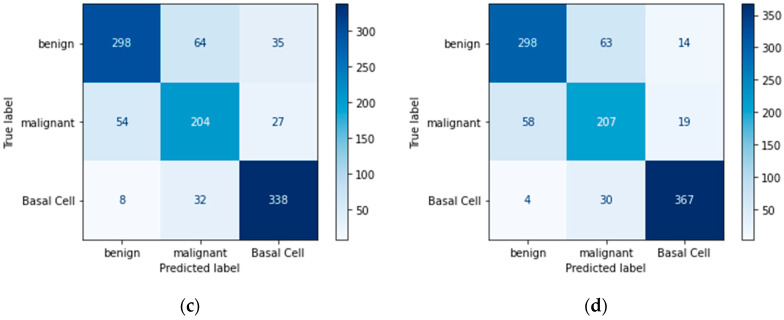
SVM confusion matrix: (**a**) imbalanced class without augmentation; (**b**) imbalanced class with augmentation; (**c**) balanced class without augmentation; (**d**) balanced class with augmentation.

In Figure 15a,c, the result of SVM without data augmentation is less accurate than the data augmented approach, as shown in Figure 15b,d.

### 4.5. Predictive Result

**Figure 16:**
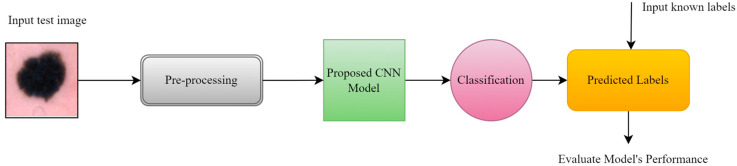
Evaluation of proposed CNN model using test data.

To measure the model’s performance, we fed some of the random test data to our proposed CNN model, with results shown in Figure 16 below.

**Figure 17:**
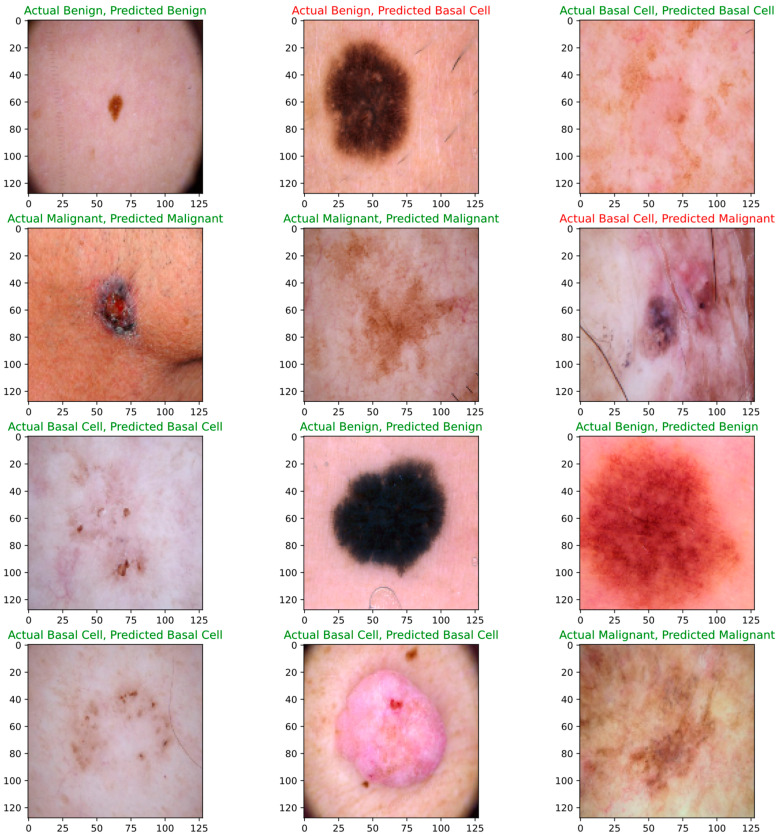
Prediction of proposed CNN model.

Figure 17 shows the output of some test samples that were provided to a trained proposed CNN model to visualize the scenario under working conditions.

### 4.6. Comparison of Proposed Method

Table 13 briefly compares the proposed method and other state-of-the-art models.

## 5. Conclusions

The study achieved an accuracy of 87.64% in diagnosing skin illnesses using a convolutional neural network (CNN), marking a significant improvement over conventional manual techniques (computer vision techniques) that are prone to human error. This research made a substantial contribution by exploring more efficient methods through machine learning, comparing shallow learning, deep learning, and hybrid models to determine the most effective approach for classifying skin diseases using dermoscopy images. Testing on ISIC archive data revealed that the CNN model, created from scratch and trained with about 8 million parameters, performed better when advanced to a more sophisticated model with seven layers of convolution and max pooling, trained with 0.6 million parameters. Under the “balanced class with data augmentation” criteria, the proposed CNN model achieved a value of 87.64%, compared to the basic CNN model’s 84.71%, Random Forest’s 84.622%, and SVM’s 84.24%. The findings indicate that the proposed model consistently outperforms others across different class imbalance combinations, with or without data augmentation.

We have performed a comprehensive literature review to know the current state-of-the-art of machine learning models in the domain of skin disease detection by utilizing the technique of PRISM, which has multiple inclusion and exclusion criteria for selecting the best papers for related research. The research goes beyond fundamental CNN models and compares the performance of the proposed model with shallow learning models such as SVM and Random Forest. This comparison provides a comprehensive assessment of the diagnostic superiority of DL. We have provided evidence through experiments that class imbalance is a big issue that can create a bias in the results. We also provided different approaches to handle class imbalance problems using data augmentation techniques.

Despite the study’s promising results, future research should apply the model to real-world healthcare. Assessing its clinical efficacy and viability is crucial for wider adoption and integration into the healthcare system. Regarding the integration of multimodal data, the study could be expanded to include clinical data or patient history for a more holistic skin condition diagnosis. Integration with other modalities may improve precision and enable individualized treatment. Deep learning models are often considered black boxes; therefore, future research could focus on interpreting and explaining their decision-making process. This would boost healthcare providers’ and patients’ trust and understanding, enabling AI-driven diagnostics.

Since deep learning models are often considered black boxes, future research could focus on interpreting and explaining their decision-making process (XAI). This would boost healthcare professionals’ and patients’ trust and understanding, enabling AI-driven diagnostics. Future dataset expansion and diversity could increase model performance and generalizability. Furthermore, the trained model can be integrated into an image-capturing device to produce a real-time classification and optimize itself with the feedback of health practitioners.

## Figures and Tables

**Table 1:** Distribution of imbalanced class: train, validation, and test sets.

Classes	Training Samples 100%	Augmented Training Samples 100%	Total Training Samples 80%	Validation Samples 20%	Test Samples
MAL	1197	3591	2873	718	300
BEN	1440	4320	3456	864	360
BCC	1125	3375	2700	675	400
Total	3762	11,286	9029	2257	1060

**Table 2:** Distribution of balanced class: train, validation, and test sets.

Classes	Training Samples 100%	Augmented Training Samples 100%	Total Training Samples 80%	Validation Samples 20%	Test Samples
MAL	1125	3375	2700	675	300
BEN	1125	3375	2700	675	360
BCC	1125	3375	2700	675	400
Total	3375	10,125	8100	2025	1060

**Table 3:** Hyperparameters selected for SVM.

Hyperparameters	Grid Search	Best Parameter
Regularization	0.1, 1, 10, 100	0.1
Gamma	0.0001, 0.001, 0.1, 1	0.0001
Kernel	rbf, poly	poly

**Table 4:** Training hyperparameters for CNN Model.

Hyperparameters	Hyperparameters Values
Mini Batch-Size	32
Learning Rate	0.001
Epochs	50, 100
Regularization Parameter	0.00001
Optimization Function	Adam
Loss Function	Categorical Cross Entropy

**Table 5:** Test accuracy of the proposed model with 100 epochs.

Approach	Proposed CNN Model
Imbalanced class without augmentation	0.7490
Imbalanced class with augmentation	0.8698
Balanced class without augmentation	0.8339
Balanced class with augmentation	0.8764

**Table 6:** Evaluation of experiments.

Approach	Basic CNN Model	Proposed CNN Model	SVM	Random Forest
Imbalanced class without augmentation	0.5594	0.8179	0.7783	0.8113
Imbalanced class with augmentation	0.8471	0.8669	0.8424	0.8462
Balanced class without augmentation	0.7962	0.7339	0.7924	0.8113
Balanced class with augmentation	0.7952	0.8688	0.8226	0.8424

**Table 7:** Test accuracy of hybrid CNN model.

Estimators	Accuracy
100	0.8160
200	0.8169
300	0.8141
400	0.8216
500	0.8198

**Table 8:** Performance metrics of basic CNN Model with different approaches.

Approach	BEN			MAL			BCC		
	PRE	REC	F-Score	PRE	REC	F-Score	PRE	REC	F-Score
Imbalanced class without augmentation	0.68	0.51	0.58	1.0	0.07	0.13	0.50	0.97	0.66
Imbalanced class with augmentation	0.86	0.80	0.83	0.75	0.80	0.77	0.92	0.93	0.92
Balanced class without augmentation	0.85	0.76	0.80	0.66	0.77	0.71	0.87	0.85	0.86
Balanced class with augmentation	0.80	0.81	0.80	0.65	0.79	0.71	0.94	0.79	0.86

**Table 9:** Performance metrics of proposed CNN model with different approaches.

Approach	BEN			MAL			BCC		
	PRE	REC	F-Score	PRE	REC	F-Score	PRE	REC	F-Score
Imbalanced class without augmentation—50 Epochs	0.78	0.82	0.80	0.74	0.80	0.77	0.92	0.83	0.88
Imbalanced class with augmentation—50 Epochs	0.87	0.84	0.86	0.77	0.84	0.80	0.95	0.91	0.93
Balanced class without augmentation—50 Epochs	0.73	0.79	0.76	0.62	0.88	0.72	0.95	0.57	0.71
Balanced class with augmentation—50 Epochs	0.86	0.86	0.86	0.79	0.81	0.80	0.94	0.93	0.93
Imbalanced class without augmentation—100 Epochs	0.70	0.83	0.76	0.66	0.77	0.71	0.93	0.66	0.77
Imbalanced class with augmentation—100 Epochs	0.87	0.83	0.85	0.76	0.83	0.80	0.96	0.93	0.94
Balanced class without augmentation—100 Epochs	0.90	0.79	0.84	0.71	0.79	0.75	0.89	0.91	0.90
Balanced class with augmentation—100 Epochs	0.91	0.79	0.84	0.78	0.87	0.82	0.93	0.96	0.95

**Table 10:** Performance metrics of SVM with different approaches.

Approach	BEN			MAL			BCC		
	PRE	REC	F-Score	PRE	REC	F-Score	PRE	REC	F-Score
Imbalanced class without augmentation	0.73	0.82	0.77	0.70	0.66	0.68	0.89	0.82	0.86
Imbalanced class with augmentation	0.80	0.86	0.83	0.78	0.71	0.74	0.93	0.93	0.93
Balanced class without augmentation	0.75	0.83	0.79	0.72	0.68	0.70	0.89	0.84	0.87
Balanced class with augmentation	0.79	0.83	0.81	0.73	0.69	0.71	0.92	0.92	0.92

**Table 11:** Performance metrics of Random Forest with different approaches.

Approach	BEN			MAL			BCC		
	PRE	REC	F-Score	PRE	REC	F-Score	PRE	REC	F-Score
Imbalanced class without augmentation	0.85	0.78	0.81	0.70	0.76	0.73	0.87	0.88	0.87
Imbalanced class with augmentation	0.86	0.77	0.81	0.73	0.80	0.76	0.92	0.95	0.94
Balanced class without augmentation	0.85	0.78	0.81	0.70	0.76	0.73	0.87	0.88	0.87
Balanced class with augmentation	0.88	0.75	0.81	0.72	0.81	0.76	0.91	0.95	0.93

**Table 12:** Basic understanding of confusion matrix.

	Actual Labels		
		Positive (1)	Negative (0)
**Predicted Labels**	Positive (1)	TP	FP
	Negative (0)	FN	TN

**Table 13:** Comparison of state-of-the-art model with the proposed model.

Paper No/Authors	Model	Dataset	Result
[18] Kemal et al.	CNN	HAM10000	Accuracy: 77% to 92.9%
[19] Shanthi et al.	AlexNet	DermNet (acne, keratosis, eczema herpeticum)	Accuracy: 85.7%, 92.3%, 93.3%, and 92.8% for acne, keratosis, eczema herpeticum, and utricaria, respectively
[21] Bajwa et al.	ResNet-152, DenseNet-161, SE-ResNeXt-101, and NASNet	DermNet and ISIC	Accuracy: DermNet: 98% ISIC: 99%
[22] Kaur et al.	DCNN	ISIC, PH2(for testing) (Melanoma and Benign)	Accuracy: 81.41% to 90.42%
[24] Rasel et al.	CNN	PH2 and ISIC	Accuracy: 97.50%
[25] Zhang et al.	U-Net	SCD, ISIC	N/A
[26] Jinnai et al.	FRCNN	5846 clinical images collected from patients	Accuracy: 86.2%
[27] Yu et al.	VGG16, Inception, Xception, MobileNet, ResNet50 and DenseNet161	HAM10000	Accuracy: DensNet: 86.5%, ResNet: 83.7%, MobileNet: 82.4%, Inception: 82.8%. Overall evaluation: 98.48%
[28] Mahbod et al.	AlexNet, VGG-16, ResNet-18, SVM classifier	ISIC	Accuracy: melanoma: 83.83% Seborrheic keratosis: 97.55%
[29] Abbas et al.	CNN	Acral Melanoma vs. Benign	Accuracy: 91.03%
Proposed Method	CNN	ISIC	Accuracy: 87.64%

## Data Availability

The ISIC archive dataset is available at https://www.isic-archive.com accessed on 1 September 2022.
